# Type of Sandwich Consumption Within a US Dietary Pattern Can Be Associated with Better Nutrient Intakes and Overall Diet Quality: A Modeling Study Using Data from NHANES 2013–2014

**DOI:** 10.1093/cdn/nzz097

**Published:** 2019-08-21

**Authors:** Yanni Papanikolaou, Victor L Fulgoni

**Affiliations:** 1 Nutritional Strategies, Paris, Ontario, Canada; 2 Nutrition Impact, Battle Creek, MI

**Keywords:** NHANES, sandwiches, diet quality, bread, shortfall nutrients, sodium

## Abstract

**Background:**

Previous research in adults has reported an association between sandwich consumers and increased daily energy, total fat, and sodium intakes and decreased dietary fiber intake. Additionally, sandwich consumers had a lower diet quality, as compared to non-sandwich consumers. However, the research failed to differentiate between the types of sandwiches consumed.

**Objectives:**

The purpose of this study was to model different sandwiches, using both whole-grain bread (WGB), enriched-grain bread (EGB), and soft taco tortillas, to examine associations with energy (kcal), nutrient intakes, and diet quality, in comparison to the typically consumed sandwich (control).

**Methods:**

Data from the NHANES 2013–2014 was used to complete the analyses in adults ≥19 years old, and USDA food composites were used to create 5 sandwich types, using WGB, EGB, or soft corn tortillas.

**Results:**

In the modeling analysis, adults consuming the soft corn tortilla taco had lower energy, as compared to those eating the typical sandwich. Total fat intakes were lower in the WGB and EGB grilled chicken/cheese/vegetable sandwiches and in the soft taco tortilla, in comparison to the control sandwich. Sodium intakes were lower in the WGB and EGB grilled chicken/cheese/vegetable sandwiches and in the soft taco tortilla, in comparison to the typical sandwich consumed. All WGB sandwich models and the soft taco tortilla had greater daily dietary fiber intakes, in comparison to the control sandwich. WGB, EGB, and tortilla sandwiches were also associated with greater intakes of shortfall nutrients. All sandwich models, except EGB with meat/cheese/vegetables, had higher diet qualities, in comparison to the control.

**Conclusions:**

The current data support the inclusion of certain WGB and EGB sandwiches/tortillas, within recommended dietary patterns, in American adults, and suggest that ingredients within a sandwich, rather than the just the bread component, can be an important contributor to overall nutrient intakes and nutrients to limit in the diet.

## Introduction

Analyses involving food sources of energy and nutrients in Americans have shown that the grain category is an important contributor of energy and nutrients in the total diet (1, 2). Grain foods, relative to energy (kcal), provided greater percentages of the under-consumed nutrients and nutrients of public health concern as defined by the 2015–2020 Dietary Guidelines for Americans (DGA) (3), including dietary fiber, folate, magnesium, calcium, and iron (4–9). When evaluating subcategories of grain foods, breads, rolls, and tortillas are also meaningful contributors (i.e., ≥10% in the diet) of dietary fiber, thiamin, folate, iron, zinc, and niacin to the American diet

(1, 2). Several of the nutrients contributed are sourced from fortified wheat flour, as mandated by the US FDA, and not related to gluten-free flours. While the FDA has recently approved folic acid to be added to corn flour (i.e., corn flour is used in the production of tortillas) to help prevent more neural tube defects among the Hispanic population (10), discretionary fortification and enrichment had not been previously approved in the US for corn flour. Therefore, corn tortillas, in comparison to wheat products, provided lower protein quality and were lower sources for iron, zinc, and vitamins A, D, E, and B12 (11).

Excluding mixed food dishes, previous analyses have examined sources of energy and nutrients from breads, rolls, and tortillas in adult females and males (2). While breads, rolls, and tortillas contributed 8.0% of sodium, 3.0% of total sugar, 3.6% of total fat, and 2.7% of saturated fat, and provided 7.6% of total energy in the daily diet, they also provided 12.4% of total dietary fiber, 14.9% of folate, 12.7% of iron, 7.9% of calcium, and 6.8% of magnesium on a daily basis (2), which are were all identified as shortfall nutrients by the 2015–2020 DGA. Additionally, breads, rolls, and tortillas contributed 15.7% of thiamin, 10.7% of niacin, 7% of riboflavin, and 6.2% of zinc daily. Similar findings were seen in adult males, such that 7.9% of sodium, 3.6% of total sugar, 3.3% of total fat, 2.5% of saturated fat, and 7.6% of total energy in the daily diet came from breads, rolls, and tortillas, along with 13.8% of total dietary fiber, 16.1% of folate, 13.2% of iron, 8.9% of calcium, 7.5% of magnesium, 16.6% of thiamin, 10.2% niacin, 7.0% riboflavin, and 6.0% of zinc per day (2). A recent sandwich consumption study in adults, using NHANES 2003–2012 data, investigated nutrient intakes and diet qualities [measured by USDA's Healthy Eating Index (HEI)] in sandwich consumers, compared to sandwich non-consumers (12). The researchers reported that sandwich consumption was associated with significantly increased energy (kcal/day), total fat, sodium, sugar, and saturated fat intakes, and reduced intakes of dietary fiber and food groups to encourage, including fruits and vegetables. Sandwich consumers also had a significantly lower overall diet quality, compared to sandwich non-consumers. Nonetheless, the investigators did not differentiate between the types of sandwiches consumed, such that all sandwiches were treated identically, whether they were a sandwich rich in saturated fat, sodium, and added sugar or a sandwich with lean protein, fiber-rich bread, and vegetables. What We Eat in America (WWEIA)/National Health and Nutrition Examination Survey 2009–2012 data have previously shown that the majority of sandwiches consumed by US adults are rich in calories, saturated fat, and sodium. Indeed, 54% of the sandwiches eaten are hamburgers, cold cuts, hot dogs/sausages [13], which can be accompanied with foods containing greater amounts of total fat, saturated fat, and sodium. Since WWEIA/NHANES data has shown that breads, rolls, and tortillas contribute approximately 8% of all calories in the US adult diet (2), and are minimal contributors of saturated fat and added sugar in the diet, it is likely that the ingredients within sandwiches are key contributors of those nutrients the 2015–2020 DGA advises should be limited and encouraged (3). Simultaneously, with the current social popularity of low-carbohydrate diets, breads in sandwiches and breads in general whether whole or enriched grain, may be perceived as lacking nutrient density and/or serve as unhealthy contributors to the American diet.

To our knowledge, there is no current, peer-reviewed study that has examined the consumption of different types of sandwiches in adults and their associations with nutrient intakes and diet quality. Sandwiches may vary widely in nutrient content and/or diet quality due to different ingredient combinations used to make the sandwich. The purpose of this study was to model different sandwiches, using both whole-grain bread (WGB), enriched-grain bread (EGB), and soft taco tortillas, to examine associations with energy (kcal), nutrient intakes, and diet qualities, in comparison to the typically consumed sandwiches in US adults.

## Methods

NHANES is a nationally representative, cross-sectional survey of US non-institutionalized, civilian residents. NHANES data are collected by the National Center for Health Statistics of the Centers for Disease Control and Prevention. Written informed consent was obtained for all participants or proxies, and the survey protocol was approved by the Research Ethics Review Board at the National Center for Health Statistics. Data from the NHANES 2013–2014 data set was used to complete the analyses in adults ≥19 years old. Nutrient intake data for NHANES 2013–2014 are from the USDA Food and Nutrient Database for Dietary Studies 2013–2014 (14), which provided the nutrient values for foods and beverages reported in WWEIA, the dietary intake component of NHANES, for each data release. The WWEIA Food Categories provide an application to analyze food and beverages, as consumed in the American diet. The classification scheme includes 150 unique categories, with are 15 main food groups and 46 subcategories of foods. The WWEIA food categories have been previously published by the USDA (15).

In the current analyses, the modeling represented theoretical sandwich compositions via formulation. The modeling exercise was meant to amplify how modifications to sandwiches can impact nutrient intakes and diet qualities. Additionally, the modeling did not include condiments; thus, the theoretical sandwiches would be lower in total energy and fat contribution in the diet, relative to typically consumed sandwiches in American dietary patterns, which are often accompanied with calorie- and fat-dense sauces and/or condiments. To develop a healthier sandwich, USDA food composites were used to create 5 sandwiches: sandwich 1 had a 2-ounce equivalent of WGB, a 2.5-ounce equivalent of meat (red meat, including beef, pork, or lamb, as defined by the USDA), a 1-cup equivalent of dairy (cheese), a 0.1-cup equivalent of dark green vegetables, and 0.1 cup of red/orange vegetables; sandwich 2 was the same as sandwich 1, but with the WBG replaced with EGB; sandwich 3 was the same as sandwich 1 (including WGB), but with the red meat replaced with grilled chicken; sandwich 4 was the same as sandwich 3, but with the WGB replaced with EGB; and sandwich 5 was a soft corn tortilla taco with chicken and cheese (as defined by USDA food codes 58,101,450, 58,101,610, and 58,101,615). For each food group (whole grain, meat, cheese, dark green vegetables, red/orange vegetables, and refined grain) the food codes that qualify and the amounts in the USDA composite were used. The food codes were weighted so that each food group in the food code contributes proportionally, as eaten in NHANES, and the sum for the food group adds to the prespecified amount for the sandwich.

Diet quality was assessed using the USDA's HEI-2010 total scores. The HEI-2010 has been developed for individuals ≥2 years of age; it provides a measure of diet quality and measures conformance to federal dietary guidance. Historically, the HEI been used to monitor dietary practices of the US population and the low-income subpopulation (16).

The NHANES data set sample included 13,799 male and female participants, ≥19 years of age, who had reliable and complete 24-hour dietary intake data from WWEIA. Trained individuals completed the 24-hour dietary recalls using the USDA's dietary data collection instrument, the Automated Multiple-Pass Method, which includes detailed descriptions of all food and food amounts consumed by subjects (17). While 2 days of 24-hour dietary recalls are collected in WWEIA, the current analysis used Day 1 data, as this represented the in-person data collection in the Mobile Examination Center (18).

All statistical analyses were performed using SAS software (Version 9.2, SAS Institute). SAS PROC SURVEYMEANS was used for all statistical calculations, including means and percentages. Survey weights were used to generate nationally representative estimates for US adults, which were also adjusted for the complex sample design of NHANES. Least square means, standard errors, and lower/upper and 99^th^ confidence levels of energy, macronutrients, and micronutrients for the daily total diet were determined with the typical sandwich (i.e., sandwiches as currently consumed and documented by NHANES in the United States) and with the USDA food code–created sandwich models. For the sandwich models, energy, nutrients, and food groups from typical sandwiches were removed from an original NHANES analysis that determined energy/nutrient intakes from all sandwiches in the US diet, and were replaced with those from the modeled sandwiches. Then least square means, standard errors, and lower/upper and 99^th^ confidence levels of energy, macronutrients, and micronutrients for the daily total diet were re-determined. When 99^th^ confidence levels did not overlap, we deemed changes meaningful. While NHANES makes available nutrients from dietary supplements, the present analysis did not include dietary supplements in the analysis, in order to focus on those nutrients gained solely from food and beverages.

## Results

The energy and nutrient profiles of the modeled sandwiches are presented in **Table 1**. Least square means for shortfall nutrients and HEI scores for typical and modeled sandwiches in the American diet can be reviewed in **Tables 2**–**6** (i.e., Sandwich 0 in Tables 2–6 represents sandwiches in the current, typical dietary pattern).

**TABLE 1:** Nutrient profile of modeled sandwiches

Energy/Nutrients	Sandwich 0^1^	Sandwich 1; Whole-Grain Bread with Meat/Cheese	Sandwich 2; Enriched-Grain Bread with Meat/Cheese	Sandwich 3; Whole-Grain Bread with Grilled Chicken/Cheese	Sandwich 4; Enriched-Grain Bread with Grilled Chicken/Cheese	Sandwich 5; Soft Corn Tortilla Taco with Chicken/Cheese
Energy, kcal	561	437	457	413	433	264
Carbohydrate, g	35	28	34	26	33	30
Protein, g	34	32	30	39	37	13
Total fat, g	28	22	22	16	16	10
Saturated fat, g	9.6	10.3	10.3	8.6	8.6	3.8
Dietary fiber, g	2.1	4.1	1.9	4.0	1.8	4.1
Iron, mg	4.7	3.1	3.8	2.0	2.8	2.4
Calcium, mg	251.2	409.7	455.2	412.7	458.2	135.8
Magnesium, mg	45.4	79.2	48.6	86.9	56.3	35.7
Potassium, mg	485	544.9	471.4	521.0	447.6	324.1
Sodium, mg	1393	1298.3	1387.5	777.0	866.2	681.8
Folate, DFE, mcg	111	54.9	129.2	47.5	121.9	98.3
Niacin, mg	7.9	6.6	6.8	10.6	10.8	3.2
Riboflavin, mg	0.5	0.45	0.51	0.34	0.40	0.18
Thiamin, mg	0.4	0.39	0.48	0.28	0.37	0.25
Vitamin A, RAE, mcg	60.1	143.1	143.1	140.5	140.5	38.8
Vitamin C, mg	3.6	4.5	4.5	4.9	4.9	2.4
Vitamin D, mcg	0.5	0.93	0.93	0.30	0.30	0.08
Vitamin E, mg	0.8	1.16	0.76	1.08	0.68	0.78

DFE = dietary folate equivalents; RAE = retinol activity equivalents.

1 Current sandwich consumed in the typical US dietary pattern.

**TABLE 2:** Least square mean intakes in the total daily diet when substituting USDA-developed sandwiches for typically consumed sandwiches

	Sandwich Number	Mean ± SE	LCL99, UCL99
Energy, kcal	0	2302 ± 12.4	2267, 2334
	1	2289 ± 11.6	2258, 2319
	2	2314 ± 11.6	2283, 2344
	3	2258 ± 11.6	2227, 2288
	4	2283 ± 11.6	2253, 2313
	5	2071 ± 11.6	2041, 2102
Total fat, g	0	89.2 ± 0.58	87.7, 90.8
	1	88.8 ± 0.56	87.3, 90.3
	2	88.8 ± 0.56	87.3, 90.3
	3	82.1 ± 0.56	80.6, 83.6
	4	82.1 ± 0.56	80.7, 83.6
	5	74.5 ± 0.56	73.0, 75.9
Saturated fat, g	0	29.5 ± 0.21	29.0, 30.1
	1	32.8 ± 0.21	32.3, 33.4
	2	32.8 ± 0.21	32.3, 33.4
	3	30.7 ± 0.21	30.2, 31.2
	4	30.7 ± 0.21	30.2, 31.2
	5	24.7 ± 0.21	24.1, 25.2
Added sugar, teaspoon equivalent	0	20.3 ± 0.26	19.6, 21.0
	1	19.9 ± 0.26	19.2, 20.5
	2	19.7 ± 0.26	19.1, 20.4
	3	19.7 ± 0.26	19.0, 20.4
	4	19.6 ± 0.26	18.9, 20.2
	5	18.9 ± 0.26	18.2, 19.6
Sodium, mg	0	3862 ± 22.8	3802, 3922
	1	4098 ± 20.8	4043, 4153
	2	4210 ± 20.9	4155, 4265
	3	3444 ± 20.6	3390, 3498
	4	3556 ± 20.6	3502, 3610
	5	3324 ± 20.7	3270, 3379

Data are from adults’ (≥19 years old; *N = *13,799) gender-combined daily intake data from NHANES 2013–2014. LCL99 = 99^th^ lower confidence level; UCL99 = 99^th^ upper confidence level.

**TABLE 3:** 2015–2020 Dietary Guidelines, Nutrients of Public Health Concern

	Sandwich Number	Mean ± SE	LCL99, UCL99
Dietary fiber, g	0	16.2 ± 0.15	15.8, 16.6
	1	18.2 ± 0.13	17.8, 18.5
	2	15.4 ± 0.13	15.1, 15.8
	3	18.1 ± 0.13	17.8, 18.4
	4	15.4 ± 0.13	15.0, 15.7
	5	18.3 ± 0.13	17.9, 18.6
Calcium, mg	0	1030 ± 8.5	1008, 1052
	1	1263 ± 7.6	1243, 1283
	2	1320 ± 7.7	1299, 1340
	3	1266 ± 7.6	1246, 1286
	4	1323 ± 7.7	1303, 1344
	5	919 ± 7.4	900, 938
Vitamin D, mcg	0	4.7 ± 0.07	4.5, 4.9
	1	5.0 ± 0.06	4.8, 5.1
	2	5.0 ± 0.06	4.8, 5.1
	3	4.2 ± 0.06	4.0, 4.3
	4	4.2 ± 0.06	4.0, 4.3
	5	3.9 ± 0.06	3.7, 4.1
Potassium, mg	0	2779 ± 18.6	2730, 2827
	1	2937 ± 17.3	2891, 2983
	2	2845 ± 17.3	2799, 2890
	3	2907 ± 17.3	2862, 2953
	4	2815 ± 17.3	2769, 2860
	5	2660 ± 17.3	2615, 2706

Least square mean dietary fiber (g), calcium (mg), vitamin D (mcg) and potassium (mg) intake in the total daily diet when substituting USDA-developed sandwiches for typically consumed sandwiches in adults’ (≥19 years old; *n = *13,799) gender-combined daily intake data from NHANES 2013–2014. LCL99 = 99^th^ lower confidence level; UCL99 = 99^th^ upper confidence level.

**TABLE 4:** 2015–2020 Dietary Guidelines Shortfall Nutrients

	Sandwich Number	Mean ± SE	LCL99, UCL99
Folate, DFE, mcg	0	556.1 ± 5.1	542.8, 569.4
	1	490.1 ± 4.9	477.1, 503.0
	2	583.3 ± 5.0	570.3, 596.3
	3	480.9 ± 4.9	467.9, 493.8
	4	574.1 ± 4.9	561.1, 587.1
	5	544.6 ± 4.9	531.6, 557.6
Iron, mg	0	16.1 ± 0.1	15.9, 16.4
	1	15.6 ± 0.1	15.3, 15.8
	2	16.5 ± 0.1	16.3, 16.8
	3	14.3 ± 0.1	14.0, 14.5
	4	15.2 ± 0.1	15.0, 15.5
	5	14.7 ± 0.1	14.5, 15.0
Magnesium, mg	0	303 ± 2.2	297, 309
	1	345 ± 2.0	340, 350
	2	307 ± 2.0	302, 312
	3	355 ± 2.0	350, 360
	4	316 ± 2.0	311, 321
	5	290 ± 2.0	285, 296
Vitamin A, RAE, mcg	0	596 ± 7.3	577, 615
	1	689 ± 6.8	671, 707
	2	689 ± 6.8	671, 707
	3	686 ± 6.8	668, 704
	4	686 ± 6.8	668, 704
	5	558 ± 6.7	541, 576
Vitamin E, mg, as alpha-tocopherol	0	8.0 ± 0.1	7.8, 8.2
	1	8.0 ± 0.1	7.8, 8.3
	2	7.5 ± 0.1	7.3, 7.8
	3	7.9 ± 0.1	7.7, 8.2
	4	7.4 ± 0.1	7.2, 7.7
	5	7.6 ± 0.1	7.4, 7.8
Vitamin C, mg	0	81.2 ± 1.5	77.4, 85.1
	1	81.6 ± 1.4	77.9, 85.4
	2	81.6 ± 1.4	77.9, 85.4
	3	82.1 ± 1.4	78.4, 85.9
	4	82.1 ± 1.4	78.4, 85.9
	5	79.0 ± 1.4	75.2, 82.7

Least square mean folate and DFE (mcg); iron (mg); magnesium (mg); vitamin A, RAE (mcg); vitamin E, as alpha-tocopherol (mg); and vitamin C (mg) intakes in the total daily diet when substituting USDA-developed sandwiches for typically consumed sandwiches in adults’ (≥19 years old; *n = *13,799), gender-combined daily intake data from NHANES 2013–2014. DFE = dietary folate equivalents; LCL99 = 99^th^ lower confidence level; RAE = retinol activity equivalents; UCL99 = 99^th^ upper confidence level.

**TABLE 5:** Least square mean thiamin (mg), riboflavin (mg), and niacin (mg) intakes in the total daily diet when substituting USDA-developed sandwiches for typically consumed sandwiches

	Sandwich Number	Mean ± SE	LCL99, UCL99
Thiamin, mg	0	1.76 ± 0.01	1.73, 1.79
	1	1.71 ± 0.01	1.68, 1.74
	2	1.82 ± 0.01	1.80, 1.85
	3	1.57 ± 0.01	1.54, 1.59
	4	1.68 ± 0.01	1.65, 1.71
	5	1.53 ± 0.01	1.50, 1.56
Riboflavin, mg	0	2.32 ± 0.02	2.27, 2.37
	1	2.37 ± 0.02	2.33, 2.42
	2	2.45 ± 0.02	2.40, 2.49
	3	2.24 ± 0.02	2.19, 2.29
	4	2.31 ± 0.02	2.27, 2.36
	5	2.05 ± 0.02	2.00, 2.09
Niacin, mg	0	27.7 ± 0.21	27.1, 28.2
	1	27.0 ± 0.17	26.5, 27.4
	2	27.2 ± 0.17	26.8, 27.7
	3	32.0 ± 0.18	31.5, 32.5
	4	32.2 ± 0.18	31.8, 32.7
	5	22.7 ± 0.17	22.3, 23.2

Data are from adults’ (≥19 years old; *n = *13,799) gender-combined daily intake data from NHANES 2013–2014; LCL99 = 99^th^ lower confidence level; UCL99 = 99^th^ upper confidence level.

**TABLE 6:** Least square mean Healthy Eating Index–2010 total score when substituting USDA-developed sandwiches for typically consumed sandwiches

	Sandwich Number	Mean ± SE	LCL99, UCL99
HEI-2010 score	0	48.7 ± 0.26	48.0, 49.4
HEI-2010 score	1	55.5 ± 0.20	54.9, 56.0
HEI-2010 score	2	46.4 ± 0.22	45.8, 47.0
HEI-2010 score	3	58.6 ± 0.20	58.0, 59.1
HEI-2010 score	4	49.3 ± 0.22	48.8, 49.9
HEI-2010 score	5	50.1 ± 0.23	49.5, 50.7

Data are from adults’ (≥19 years old; *n = *13,799) gender-combined daily intake data from NHANES 2013–2014. HEI = Healthy Eating Index; LCL99 = 99^th^ lower confidence level; UCL99 = 99^th^ upper confidence level.

### Energy and total fat intake: Added sugars, sodium, and saturated fat

The energy intake from the total daily diet was approximately 2302 ± 12.4 kcal/day. Substitutions of the 5 sandwiches for the typical sandwiches showed that only the soft taco with chicken and cheese was associated with lower daily calories when compared to the current sandwich, such that consuming a soft taco was associated with a reduction of 230 kcal per day (2071 ± 11.6 for soft taco vs 2302 ± 12.4 for typical sandwich). No meaningful differences were observed when comparing the WGB and EGB sandwiches to current sandwiches consumed (Table 2).

The total fat intake from the total daily diet was approximately 89 g. Substitutions of the 5 sandwiches for the typical sandwiches showed that WGB and EGB grilled chicken/cheese sandwiches and soft taco with chicken/cheese were associated with reduced total fat intakes, compared to the typical sandwich. No meaningful differences were observed for total fat intakes between the WGB and EGB meat/cheese sandwiches and the typical sandwich. Modeling the soft taco tortilla with chicken/cheese resulted in less total fat intake, in comparison to all other models examined (Table 2).

Based on current consumption in the American diet, added sugar intake from the total daily diet was 20.3 ± 0.26 teaspoon equivalents. The soft taco tortilla with chicken/cheese was associated with a lower daily added sugar intake when compared to the current sandwich, such that consuming a soft taco with chicken was associated with approximately a 1.5-teaspoon equivalent less of added sugar (18.9 ± 0.26 teaspoon equivalent for soft taco vs 20.3 ± 0.26 teaspoon equivalent for typical sandwich). No other meaningful differences were observed when comparing the WGB and EGB sandwiches to current sandwiches consumed (Table 2).

The sodium intake from the total daily diet was approximately 3862 mg/day. Sodium reductions by sandwich-type substitutions ranged from 306 to 538 mg/day. The WGB grilled chicken/cheese sandwich, the EGB grilled chicken/cheese sandwich, and the soft taco were associated with lower sodium intakes, relative to the current sandwich. In contrast, both the WGB meat/cheese and the EGB meat/cheese sandwiches were associated with greater daily sodium intakes, in comparison to the current sandwich. Sodium intakes were significantly reduced when consuming WGB and EGB chicken/cheese sandwiches and soft tacos, when compared to WGB and EGB meat/cheese sandwiches (Table 2).

The saturated fat intake from the total daily diet was approximately 29.5 g/day. The soft taco tortilla with chicken and cheese was associated with a lower daily saturated fat intake, compared to the current sandwich (24.7 ± 0.2 vs 29.5 ± 0.2 g/day, respectively). All 4 other sandwich substitutes with WGB and EGB were associated with greater daily saturated fat intakes, relative to the current sandwich, likely resulting from the meat ingredients within the sandwiches (Table 2).

### Nutrients of public health concern

The dietary fiber intake from the total daily diet was approximately 16.2 g/day. The WGB meat/cheese sandwich, WGB and EGB grilled chicken/cheese sandwiches, and the soft taco with chicken/cheese dietary patterns were all associated with higher dietary fiber intakes when compared to the current sandwich, whereas no meaningful associations were seen with the EGB meat/cheese sandwich, relative to the typical sandwich. Consumption of the EGB chicken/cheese sandwich indicated a lower dietary fiber intake, when compared to the typical sandwich consumed (Table 3).

Prior to the sandwich substitutions, an analysis of NHANES showed the calcium intake from the total daily diet to be approximately 1030 mg/day. All sandwich substitutions with WGB and EGB were associated with greater calcium intakes, compared to the current sandwich, with intakes ranging from 233–293 mg/day greater calcium via manipulation of the sandwich type. The soft taco with chicken/cheese substitution was associated with a lower calcium intake, compared to the current sandwich. Additionally, EGB sandwich models were associated with greater calcium intakes when compared to WGB sandwich models (Table 3).

The vitamin D intake from the total daily diet was approximately 4.7 mcg. WGB and EGB meat/cheese sandwiches were not associated with meaningful differences in vitamin D intakes, relative to sandwiches in the typical dietary pattern. WGB and EGB grilled chicken/cheese sandwiches and soft tacos with chicken and cheese were associated with lower vitamin D intakes, versus the typical sandwich. Both WGB and EGB meat/cheese sandwiches were associated with greater vitamin D intakes, in comparison to the WGB/EGB grilled chicken and soft taco sandwiches (Table 3).

The daily potassium intake from the total diet was approximately 2779 mg. WGB meat/cheese and WGB grilled chicken/cheese were associated meaningfully greater potassium intakes, in comparison to sandwiches in the typical dietary pattern, with no meaningful differences observed between EGB meat/cheese and grilled chicken/cheese sandwiches, relative to typically consumed sandwiches. Modeling the soft taco consumption was associated with a lower potassium intake, versus the typical sandwich. The potassium intakes were greater when modeling WGB sandwiches, in comparison to the EGB sandwiches (Table 3).

### Shortfall nutrients

The folate and dietary folate equivalents (DFE) intake from the total daily diet was approximately 556 mcg/day. Both versions of the WGB sandwich were significantly associated with a lower folate and DFE intake, when compared to the current sandwich. In contrast, substituting either EGB sandwich for the current sandwich was associated with an increased dietary folate intake (Sandwich 2: 583 ± 5.0 vs Sandwich 0: 556 ± 5.1 mcg/day; Sandwich 4: 574 ± 4.9 vs 556 ± 5.1 mcg/day). Both the EGB meat/cheese and grilled chicken sandwiches were associated with a greater folate and DFE intake, relative to both WGB sandwiches. No meaningful differences were seen between the soft taco tortilla and the typical sandwich (Table 4).

The iron intake from the total daily diet was approximately 16.1 mg/day. Both versions of the WGB sandwiches were associated with a lower iron intake, relative to the current sandwich. Modeling of the EGB meat/cheese sandwiches was associated with greater iron intake, as compared to the WGB meat/cheese sandwiches, while modeling of the consumption of the EGB grilled chicken/cheese was associated with greater iron intake, versus the WGB grilled chicken/cheese. Modeling of the soft taco with chicken and cheese was not associated with differences versus the EGB grilled chicken/cheese sandwich, but showed a lower iron intake when compared to the EGB meat/cheese sandwich (Table 4).

The magnesium intake from the total daily diet was approximately 303 mg/day. No meaningful differences were seen when comparing the EGB meat/cheese sandwich with the current sandwich. In contrast, modeling of several sandwich types was associated with a greater magnesium daily intake. Specifically, the WGB meat/cheese, WGB grilled chicken/cheese, and EGB grilled chicken/cheese sandwiches were associated with greater magnesium intakes, versus the sandwiches in the typical dietary pattern. Modeling of the soft taco with chicken/cheese was not associated with a meaningful difference, in comparison to the current sandwich. In general, the consumption of WGB sandwiches was linked to a greater daily magnesium intake, relative to the consumption of EGB sandwiches (Table 4).

The vitamin A intake from the total daily diet was approximately 596 retinol activity equivalents mcg/day. Both versions of the WGB sandwiches and the EGB sandwiches were associated with greater vitamin A intakes, compared to the current sandwich, with no meaningful differences seen between WGB and EGB sandwiches. The consumption of a soft taco with chicken/cheese was associated with a lower vitamin A intake, relative to all other sandwich models (Table 4).

The vitamin E as alpha-tocopherol intake from the total daily diet was approximately 8 mg/day. No associations were seen when comparing the current sandwich to the WGB meat/cheese sandwiches. The WGB sandwiches were associated with greater vitamin E, relative to the EGB sandwich models, with no meaningful differences seen between EGB sandwiches and the soft taco tortilla model (Table 4).

The vitamin C intake from the total daily diet was approximately 81 mg/day. No meaningful differences in intake were seen when comparing the current sandwich to any of the sandwich models examined (Table 4).

### Enrichment nutrients

The thiamin intake from the total daily diet was approximately 1.8 mg/day (Table 5). Both versions of the EGB sandwich were associated with a greater thiamin intake, relative to the WGB sandwich models and the soft taco with chicken/cheese model. The soft taco tortilla model was associated with a reduced thiamin intake, relative to the typical current sandwich.

The riboflavin intake from the total daily diet was approximately 2.3 mg/day (Table 5). No meaningful differences were seen when comparing the WGB meat/cheese and EGB meat/cheese sandwiches. Similarly, no meaningful differences were observed when comparing the WGB grilled chicken and EGB grilled chicken sandwiches. The soft taco with chicken/cheese was associated with lowered riboflavin, relative to all other sandwich models.

The niacin intake from the total daily diet was approximately 27.7 mg/day (Table 5). No meaningful differences were observed between the current sandwich and the WGB and EGB meat/cheese sandwiches. In contrast, both the WGB and EGB grilled chicken sandwich models were associated with a greater niacin intake, relative to the current sandwich and to the WGB/EGB meat/cheese sandwich models. The soft taco model was associated with a niacin intake value that was lower than the other models considered.

### Diet quality: Healthy Eating Index 2010

The HEI 2010 score for the total daily diet was approximately 48.7 out of 100 (Table 6). Both WGB sandwich models, in addition to the soft taco, were associated with higher HEI scores, relative to the typical sandwich. Consuming EGB meat/cheese sandwich was associated with a lower diet quality score, compared to the typical sandwich pattern, while no meaningful difference was observed when comparing the EGB grilled chicken/cheese and the typical sandwich pattern. Modeling of the WGB grilled chicken indicated a greater diet quality score, when compared to the WGB meat/cheese sandwich.

## Discussion

While previous studies have conducted analyses involving sandwich consumption, the analyses have not differentiated between or considered types of sandwiches. To our knowledge, this is the first study that has modeled various types of WGB and EGB sandwiches within the US adult dietary pattern. The current study showed that WGB, EGB sandwiches, and soft taco sandwiches were associated with several nutrient intake benefits, when compared to the typically consumed sandwich. WGB and EGB grilled chicken/cheese sandwiches and the soft taco with chicken and cheese were associated with reduced total fat intakes, compared to the typical sandwich, with no meaningful differences for total fat intakes between the WGB and EGB meat/cheese sandwiches and the typical sandwich. Modeling of the soft taco with chicken and cheese resulted in a meaningfully lower total fat intake, in comparison to all other models examined, thus illustrating that the ingredients within a sandwich can be key drivers to the daily total fat intake, rather than the type of bread product used to create the sandwich. Similar findings were observed when evaluating those nutrients the 2015–2020 DGA advised limiting (added sugars, sodium, and saturated fat).

While sandwiches have been identified by WWEIA as a mainstay of the American diet (12, 13), limited evidence is available in the published scientific literature examining sandwich consumption as part of an adult dietary pattern. Nearly half of all adults in the US consume 1 or more sandwiches on any given day, with the most commonly consumed types of sandwiches being cold-cut meat sandwiches, burgers, poultry, and hot dog/sausage sandwiches. Moreover, it has previously been reported that adults who consume sandwiches consume an additional 278 kcal, relative to adults who do not consume sandwiches. It has also been reported that sandwiches substantially contribute to energy intake, nutrients, and MyPlate food groups (13). Data from WWEIA 2009–2012 showed that sandwiches provide nutrients that are considered to have shortfalls and/or nutrients of public health concern. The former includes 12% of folate, 13% of iron, 9% of magnesium, and 8% of vitamin E, while that latter includes 10% of vitamin D, 15% of all calcium, and 9% of dietary fiber and potassium in the total US adult diet. When considering nutrients to limit, sandwiches also contribute to 18% of daily sodium and 17% of saturated fat in the total diet (13).

An et al. (12) hypothesized that sandwich consumption can profoundly impact an individual's caloric intake and diet quality. Using data from NHANES 2003–2012, all adult sandwich consumers were identified and compared against sandwich non-consumers with respect to energy, nutrient intakes, and diet qualities. Results showed that, relative to non-consumption, sandwich consumption was significantly associated with increased total daily calories (∼100 kcal), total fat (∼7 g), and sodium (∼270 mg) and a reduced HEI diet quality score. However, an important limitation of the study was that sandwiches were not differentiated: sandwiches of every type—irrespective of calories, total fat, saturated fat, added sugar, and sodium—were included in the analysis. Our current modeling analysis demonstrated that substitutions of sandwich ingredients for food items that are lower in total fat, saturated fat, added sugars, and sodium can have meaningful and positive influences on daily energy and nutrient intakes, independent of whether WGB, EGB, or soft taco tortillas are incorporated. Our analysis also showed that fiber intakes were higher when WGB was modeled, thus suggesting that industry innovations may need to increase the fiber content per serving of EGB.

The present modeling analysis includes study limitations characteristic of observational research, which have been documented in prior publications (19). The results for the typically consumed sandwiches and dietary patterns are dependent on self-reported dietary data for foods from NHANES, which may involve study participants under- or over-estimating their food consumption, leading to inaccuracies in energy and nutrient intakes. Data were also obtained using a 24-hour dietary recall, which relies on the study participant's memory and, while validated methods were used to gather the data, recall information is subject to inaccuracies and bias from memory challenges and from other potential measurement errors experienced in epidemiological investigations using large data sets (19). Nonetheless, an important strength of NHANES includes a large and nationally representative data set of adults in the United States, and corresponding food and nutrient intake data (20, 21). The sandwich models used in the present analysis did not incorporate sauces and/or condiments, which are typically consumed in the American diet with sandwiches. Since sandwich consumption remains a major component of the US typical dietary pattern, future research recommendations include modeling different sandwich types, with a focus on ingredients and portion sizes. In addition, modeling sandwich consumption in children and examining nutrient intakes and diet qualities, as part of future research, will further contribute and advance the nutritional literature.

Modeling different types of sandwich consumption in the US adult diet, using WGB, EGB, and soft taco tortillas, was associated with several nutrient intake benefits, when compared to the typically consumed sandwich. Additionally, WGB, EGB, and soft taco tortillas can contribute to the nutrient density of an adult dietary pattern, since WGB, EGB, and tortilla sandwiches were also associated with greater intakes of shortfall nutrients. Likewise, most sandwich models were associated with a higher diet quality, in comparison to the typical sandwich. Overall, the current data support the inclusion of certain WGB and EGB sandwiches and tortillas within recommended dietary patterns in American adults, and suggest that ingredients within a sandwich, rather than the just the bread component, can be important contributors to overall nutrient intakes and nutrients to limit in the American diet.

## Notes

This research was supported by the Grain Foods Foundation, Washington, DC.

Author disclosures: YP, as Vice-President of Nutritional Strategies, provides food, nutrition, and regulatory affairs consulting services for food and beverage companies and food-related associations. YP and VLF collaborate on NHANES analyses. VLF, as Senior Vice-President of Nutrition Impact, provides food and nutrition consulting services for food and beverage companies. VLF conducts analyses of NHANES data for members of the food industry.

The sponsors (Grain Foods Foundation) had no role in the design of the study or in the collection, analyses, or interpretation of the data.

Abbreviations used: DFE, dietary folate equivalents; DGA, Dietary Guidelines for Americans; EGB, enriched-grain bread; HEI-2010, Healthy Eating Index-2010; WGB, whole-grain bread; WWEIA, What We Eat in America.
